# The effect of pro-inflammatory cytokines on the discharge rate of vagal nerve paraganglia in the rat

**DOI:** 10.1016/j.resp.2010.03.001

**Published:** 2010-04-30

**Authors:** Brian Mac Grory, Edward T. O’Connor, Ken D. O’Halloran, James F.X. Jones

**Affiliations:** School of Medicine and Medical Science, Health Sciences Centre, University College Dublin, Belfield, Dublin 4, Ireland

**Keywords:** Superior laryngeal nerve, Paraganglia, Cytokine, IL-1β, TNF-α, Inflammation, Hypoxia, Carotid body, Vagus nerve

## Abstract

Vagal paraganglia resemble the carotid body and are chemosensitive to reduction in the partial pressure of oxygen ($P_{{\text{O}}_{2}}$) (O’Leary et al., 2004). We hypothesised that they may also mediate communication between the immune system and the central nervous system and more specifically respond to the pro-inflammatory cytokines: interleukin-1 beta (IL-1β) and tumour necrosis factor-α (TNF-α). We recorded axonal firing rate of isolated superfused rat glomus cells – located at the bifurcation of the superior laryngeal nerve – to IL-1β or TNF-α at concentrations of 0.5 ng/ml, 5 ng/ml and 50 ng/ml. Twenty-three successful single fibre recordings were obtained from 10 animals. IL-1β and TNF-α had no statistically significant effect on the frequency of action potentials observed (*p* = 0.39 and 0.42, respectively, repeated measures ANOVA). The activity of both cytokines was tested by observing translocation of P65-NFκB from cytoplasm to nucleus in cultured HELA cells. In conclusion, an immune role for SLN paraganglia has not been established.

## 1 Introduction

Paraganglia of the tenth cranial nerve are studded along its course with dense aggregations in the thorax and near the stomach (Coleridge et al., 1970; Howe et al., 1981). O’Leary et al. (2004) established the chemosensitive properties of these paraganglia by studying the response of the superior laryngeal nerve (SLN) to low $P_{{\text{O}}_{2}}$ and sodium cyanide (CN) in the rat. The history of peripheral arterial chemoreceptor research is dominated by studies on oxygen sensing (Daly, 1997). However, a number of recent studies have examined the role of inflammatory mediators in arterial chemoreceptors (Goehler et al., 1999; Shu et al., 2007; Lam et al., 2008). We proposed an immune role for vagal paraganglia based on their ubiquitous distribution, their proximity to potential portals of bacterial ingress in the airways and gut and the work of Shu et al. (2007) which provided evidence that the carotid body (a large paraganglion of the ninth cranial nerve) is responsive to local application of the pro-inflammatory cytokine interleukin-1 beta (IL-1β).

We developed a convenient *in vitro* preparation for studying the vagal paraganglia. In light of the enormous variety of potential stimuli that could theoretically elicit a response we wanted to develop a robust, convenient, reproducible method. We validated our preparation by replicating the previous findings of O’Leary et al. (2004). Furthermore, we used this method to assess the effect of IL-1β and tumour necrosis factor-alpha (TNF-α) on the firing rate of the vagal paraganglia. These cytokines were chosen because of their pleiotropic nature and their established role in the immune response.

Our hypothesis was that the vagal paraganglia can sense increased plasma concentrations of pro-inflammatory cytokines and, through an increase or decrease in action potential frequency, encode inflammatory stimuli as electrical events. This could, in theory, facilitate communication between the immune system and the central nervous system and form the afferent arc of the “inflammatory reflex” enunciated by Tracey (2002).

## 2 Methods

Twenty-three female Wistar rats weighing 200–300 g were killed humanely by a blow to the head followed by section of the cervical spinal cord in accordance with local institutional guidelines. Only personnel trained and experienced in this procedure were permitted to kill the animals.

**Fig. 1:**
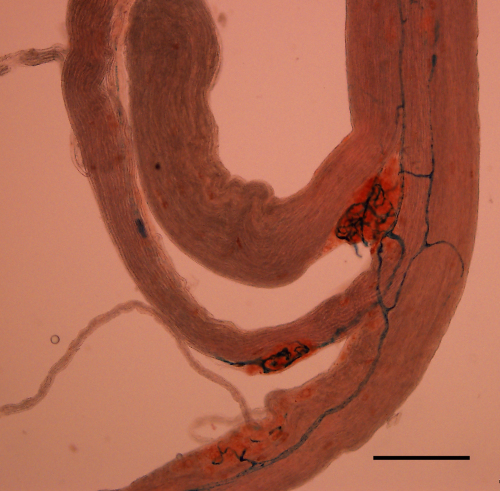
Paraganglia of the superior laryngeal nerve of the rat. The vital dye Neutral Red is selectively taken up by glomus and neuronal tissue and helps highlight the size and distribution of these structures on the SLN. The vasculature is stained with Berlin Blue. Scale bar = 200 μm.

**Fig. 2:**
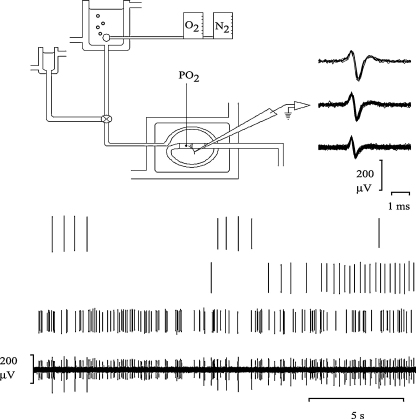
Schematic representation of the experimental apparatus. The experimental apparatus consisted of a superfusion system with a main and ancillary reservoir (of HEPES-buffered Tyrode's at 37 °C) feeding into an isolated polyvinylchloride tube, via a three-way tap. A freshly dissected SLN was partially placed in the lumen of the tubing and this was further immersed in filtered, HEPES-buffered Tyrode's at 37 °C held in a jacketed organ bath. The $P_{{\text{O}}_{2}}$ of the main reservoir could be modulated by rotameters connected to oxygen and nitrogen cylinders. The ancillary reservoir was used to deliver the CN challenges and the cytokines (IL-1β or TNF-α at concentrations of 0.5 ng/ml, 5 ng/ml or 50 ng/ml). The SLN action potentials were detected by way of a glass suction electrode, digitised and analysed by spike recognition software. The lower panel illustrates the method by which 3 individual units are discriminated by the software – based on amplitude and shape.

One of the SLNs was located and dissected free, to include its bifurcation, an area known to contain a large density of glomus tissue (Fig. 1). The nerve was then transferred to an organ bath containing warm (37 °C) HEPES-buffered Tyrode's solution (Sigma code: T2145; containing in mM NaCl 137.0, KCl 2.7, MgCl_2_ 1.0, CaCl_2_ 1.36, Na_2_HPO_4_ 0.35, (d)-glucose 5.5, HEPES 10, at pH 7.4). The experimental set-up is depicted in Fig. 2. In brief, the peripheral end of the nerve was placed in flexible polyvinyl chloride tubing (outer diameter: 5 mm; inner diameter: 3 mm) – via a small perforation/narrow opening created with a scalpel – with the proximal end exposed to the bath milieu and the bifurcation (with glomi) suspended in the lumen of the tube. Care was taken to avoid damaging the tissue and a seal was maintained by the elastic recoil of the tube and bolstered with Vaseline. The rubber tubing was superfused at 1–2 ml/min with HEPES-buffered Tyrode's solution via two reservoirs; both contained heated HEPES-buffered Tyrode's solution. O_2_ or N_2_ were bubbled through the first reservoir and IL-1β, TNF-α or CN added to the second reservoir according to experimental protocols. The reservoirs fed the tube by gravity and were connected via a three-way tap to a single inflow tube ensuring a constant and equal flow rate from both reservoirs. The $P_{{\text{O}}_{2}}$ of the superfusate was measured continuously with a $P_{{\text{O}}_{2}}$ probe (World Precision Instruments; OXEL-1 ISO_2_) and in later experiments $P_{{\text{O}}_{2}}$ and temperature were measured continuously using an oxylite 2000 system (Oxford optronix). It proved important to ensure that the fluid in the tubing dead space (which lost heat and gases) was filled with warm solution immediately before each trial. The effect of perfusing the preparation with stagnant cool solution can be seen in Fig. 4, where the first CN perfusion causes a transient dip in the $P_{{\text{O}}_{2}}$ recording. The subsequent CN tests are less affected because the solution in the tubing more closely approximates the reservoir solution.

The free end of the nerve was stripped of epineurium and single fibre recordings were made from the main trunk using a glass suction electrode (WPI; PG61150-4, internal tip diameter: 10–50 μm). The electrical signal was digitised, using an AD converter (Cambridge Electronic Design, 1401) and analysed, using spike recognition software (Spike2, Cambridge Electronic Design), which allowed discrimination of individual units from multi-unit nerve recordings (Fig. 2). The unitary nature of discriminated recordings was determined by noting the relative constancy of spike shape and amplitude by spike superimposition.

Two blocks of electrophysiological experiments were performed:

1. *Descriptive chemosensitivity experiments*: we demonstrated the versatility, reliability and reproducibility of the experimental preparation by performing chemosensitivity studies with this tissue, exposing the glomus cells to CN and different levels of $P_{{\text{O}}_{2}}$.
2. *Quantitative experiments with pro-inflammatory cytokines*: we measured the response of units to increasing concentrations of pro-inflammatory cytokines in normoxia. Only those units that were responsive to ultimate CN administration were included in analysis. Spike counts were performed on 2 min blocks of time: first, the baseline action potential count at the beginning of the experiment, then 30 s after administration of IL-1β, TNF-α or CN to 90 s after its cessation.

### 2.1 Histology

We used the histological dyes Neutral Red and Berlin Blue to stain the glomus tissue and its vasculature, respectively (Fig. 1). The whole body perfusion method was used. After inducing surgical anaesthesia with sodium pentobarbital [Euthatal] (60 mg kg^−1^ i.p.), an 18 G needle was inserted into the left ventricle and the following solutions (warmed to 37 °C) were perfused in succession: (1) 100 ml of normal saline in order to wash out the vasculature followed by (2) 100 ml of Neutral Red (1%) in normal saline then (3) 100 ml Berlin Blue (0.1%) in normal saline.

### 2.2 Immunocytochemistry

The efficacy of the cytokines was tested on cultured HELA cells exposed for 30 min to either 5 ng/ml or 10 ng/ml of IL-1β and TNF-α. The translocation of P65-NFκB from cytoplasm to nucleus was used as a marker for cytokine receptor activation. Twelve plates of cells were used in total. An antibody directed against rat P65-NFκB (Cat # SC-7151, Santa Cruz Biotechnology) was used at a concentration of 1:500 in a staining protocol identical to that described above. This was followed by a PBS rinse and a 10 min incubation step with Hoechst blue, a nuclear stain (0.5 μg/ml) (Cat # H6024, Sigma–Aldrich).

### 2.3 Analysis

The spike counts were normally distributed and the group variances were equal (Bartlett's test). Therefore, it was deemed appropriate to apply repeated measures ANOVA to test for significance of the effect of cytokine concentration. The criterion for statistical significance was *p* < 0.05. All results are expressed as mean ± SEM.

## 3 Results

**Fig. 3:**
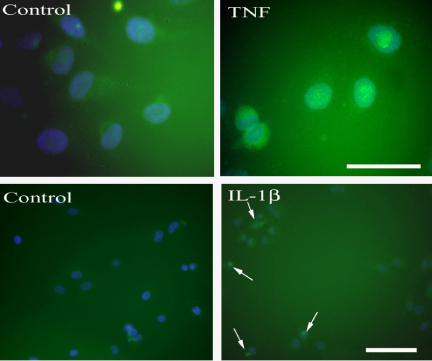
Effect of cytokines on translocation of P65-NFκB to the nuclei of HELA cells. *Upper panels*. On the left are shown control HELA cells that display a cytoplasmic location of P65-NFκB. Following 30 min exposure to TNF-α (10 ng/ml) there is an obvious translocation of P65-NFκB (green signal) to the nucleus (stained with Hoescht blue). Scale bar = 50 μm. *Lower panels*. Untreated control cells are shown on the left and the right panel shows HELA cells that were incubated with 5 ng/ml IL-1β for 30 min. The arrows indicate cells that have nuclear localization of P65-NFκB. The intense small dots are within the nuclei and may indicate the position of the nucleoli which are targets for P65-NFκB. Scale bar = 100 μm. (For interpretation of the references to color in this figure legend, the reader is referred to the web version of the article.)

Both cytokines produced the expected translocation of P65-NFκB from cytoplasm to nucleus in HELA cells (Fig. 3) when dissolved in the solutions used in our experiments.

**Fig. 4:**
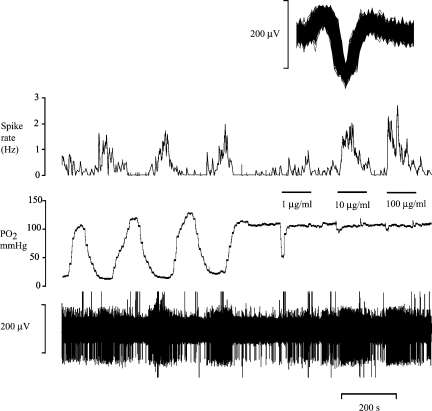
The effect of changes in oxygen partial pressure ($P_{{\text{O}}_{2}}$) on action potential frequency in the SLN. The bottom reading is a raw digitised recording, the middle trace is $P_{{\text{O}}_{2}}$ of the perfusate at a constant temperature of 37 °C. The top trace is the mean action potential frequency of a single unit (averaged over 10 s) extracted from the raw recording. The inset shows all superimposed action potentials of the discriminated single unit (window is 2 ms in duration). At the intervals indicated by horizontal bars increasing concentrations of sodium cyanide are administered to the glomi.

**Fig. 5:**
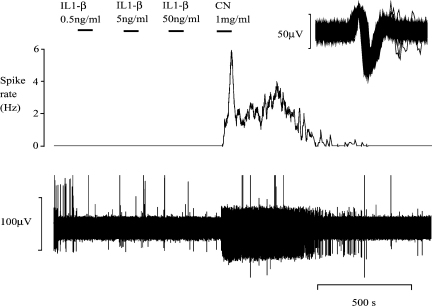
A representative experiment illustrating the effect of IL-1β on action potential frequency in the SLN. The bottom reading is a raw, digitised recording. The top trace illustrates the mean action potential frequency of a single unit (averaged over 10 s) and is derived from the raw recording. This graph is typical of the cytokine group of experiments. Application of increasing concentrations (low to high) of IL-1β does not alter the firing frequency of the SLN. The vigorous response to sodium cyanide (CN) elicited at the end of the protocol confirms the responsivity of the preparation.

Placement of nerve-attached glomi into a sealed, isolated, perfused tube led to a simple, rapid and reliable method of regulating tissue $P_{{\text{O}}_{2}}$. Fig. 4 shows the clear discharge rate changes that accompany alterations of the $P_{{\text{O}}_{2}}$ in the superfusate. CN evoked a brisk response and sustained discharge (Figs. 4 and 5).

**Fig. 6:**
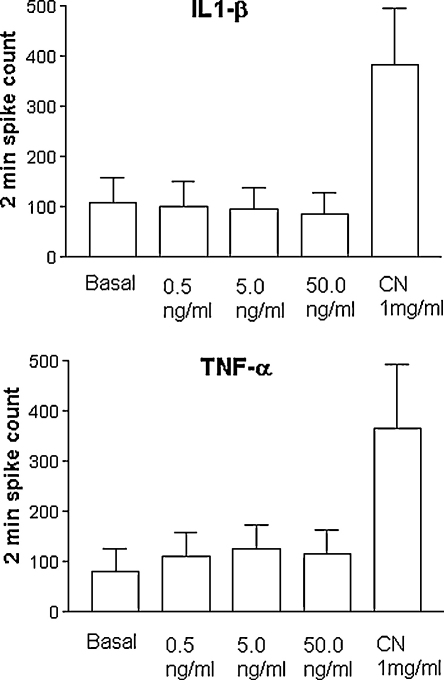
Group data of the effects of IL-1β and TNF-α on the action potential firing frequency in the SLN. The upper graph illustrates the action potential frequency with increasing concentrations of IL-1β (*n* = 14) and application of high-concentration sodium cyanide (CN). The lower graph depicts the same information with respect to TNF-α (*n* = 9). In both cases, there is no statistically significant difference between action potential frequency at baseline and at any cytokine concentration.

Our experiments showed that in an acute setting, the application of 0.5 ng/ml, 5 ng/ml and 50 ng/ml IL-1β or TNF-α evoked no statistically significant change in the frequency of action potentials: in the case of IL-1β (*n* = 14), the baseline 2-min action potential count was 108 ± 50, upon application of 0.5 ng/ml it was 100 ± 50, with 5 ng/ml it was 95 ± 44, and with 50 ng/ml it was 87 ± 42. The differences observed were not statistically significant (Fig. 6, *p* = 0.39 repeated measures ANOVA). In the case of TNF-α (*n* = 9), the baseline 2-min action potential count was 82 ± 46, upon application of 0.5 ng/ml it was 110 ± 50, with 5 ng/ml it was 126 ± 46, and with 50 ng/ml it was 115 ± 47. Again, the differences observed were not statistically significant (Fig. 6, *p* = 0.42 repeated measures ANOVA). These cytokine experiments were conducted with solutions equilibrated with room air; the atmospheric pressure was 758 ± 8 mm Hg (mean ± S.D.; values measured over 14 days).

## 4 Discussion

Paraganglia of the tenth cranial nerve are chemosensitive (Brophy et al., 1999). We have underlined and extended the previous findings of O’Leary et al. (2004) by showing that glomi are consistently found at the bifurcation of the SLN (Fig. 1). In addition, firing frequency increases in response to hypoxia and decreases in response to hyperoxia. CN inhibits complex IV, the cytochrome oxidase complex of the electron transport chain and induces a state of histotoxic hypoxia (Hargreaves et al., 2007). We used high concentrations of CN only at the end of our protocol to ensure that we still held the single unit and that the unit was chemosensitive.

One of the problems encountered by O’Leary et al. (2004) was that their open bath approach did not lend itself to rapid or large reductions in the $P_{{\text{O}}_{2}}$ of the superfusate. In their set of experiments, they managed to achieve a $P_{{\text{O}}_{2}}$ of below 60 mm Hg in only two cases. By placing the glomi in a low-volume sealed compartment we were able to generate very low $P_{{\text{O}}_{2}}$ (by saturating the superfusate with nitrogen gas) or high $P_{{\text{O}}_{2}}$. Thus, without compromising the structural integrity of the glomus cells, our method can be readily used for experiments assessing O_2_ sensitivity and CN challenges. This application of a perfused tube with a retractable slit in combination with the easy accessibility of the SLN means that this very simple preparation can be set up in under 5 min.

It appears that both cytokines tested in this study have no role in acutely changing the discharge rate of the vagal paraganglia *in vitro*. We used concentrations of 0.5 ng/ml, 5 ng/ml and 50 ng/ml for 3 reasons: first, extreme sepsis in mammals leads to plasma IL-1β concentrations of 0.5 ng/ml; second, concerns over bioavailability of the proteins led us to test very high concentrations and; third, Shu et al. (2007) used these concentrations in their study of the carotid body. The bioactivity of the cytokines in the current study was tested on HELA cells and established that our reagents interact with cytokine receptors and produce activation of the NFκB pathway.

The present study on cytokines and arterial chemoreceptors was motivated by a number of past studies. Goehler et al. (1999) demonstrated the presence of IL-1β receptors on abdominal vagal paraganglia and Shu et al. (2007) showed that IL-1β acutely increases the rate of discharge of glossopharyngeal afferents from the glomus cells of the carotid body. As the carotid body has obvious functional and morphological parallels with the paraganglia of the tenth cranial nerve, it may appear surprising that the two cytokines tested in the present study have no effect. However, the reflex actions of vagal paraganglia can differ from those of the carotid body (Jones and Daly, 1997) so there may be differential modulation of chemoreceptor function by cytokines.

Hypoxia is a pro-inflammatory state (Alam et al., 2007). The paraganglia of the tenth cranial nerve hypertrophy in response to chronic hypoxia (Dahlqvist et al., 1987). It may be that under normal conditions pro-inflammatory cytokines have no functional effect on these cells but that under conditions of chronic hypoxia, the glomi become sensitised to them. Lam et al. (2008) demonstrated that the carotid body from rats that were made chronically hypoxic powerfully up regulated the expression of cytokines and their receptors. In agreement with this, Liu et al. (2009) showed that chronic hypoxia caused increased pro-inflammatory cytokine expression in the rat carotid body, with these increases evident in both native carotid body cells as well as in the large number of invading macrophages. The chronic exposure to hypoxia made the carotid bodies more sensitive to hypoxia and this adaptation, along with the increased cytokine expression, was blocked by anti-inflammatory medication. This is tentative evidence that pro-inflammatory cytokines may contribute to the development of chemoreceptor hypersensitivity. When the cat carotid body is inflamed by i.v. injection of bacterial lipopolysaccharide it mediates the resultant tachypnoea (Fernandez et al., 2008). In this very interesting study these authors also found that TNF-α did not affect the basal firing of carotid body afferents but had an inhibitory action on hypoxia evoked discharge. Our experiments are rather limited in comparison because only basal chemoreceptor discharge was analysed.

In contrast to the accumulated evidence for carotid body glomus cell expression of IL-1 and TNF receptors, it is not known whether vagal paraganglia or vagal afferent axons express receptors for these cytokines. The latter possibility is worth exploring because it may underpin vagal dependent effects of IL-1β or TNF-α administration. Yu et al. (2007) have show that vagal pulmonary C-fibres are excited by IL-1β and therefore vagal axons may express the IL-1 receptor. To summarise, SLN paraganglia demonstrate no acute change in firing frequency in response to the application of the pro-inflammatory cytokines IL-1β and TNF-α.
